# Synergistic antitumor interaction between valproic acid, capecitabine and radiotherapy in colorectal cancer: critical role of p53

**DOI:** 10.1186/s13046-017-0647-5

**Published:** 2017-12-06

**Authors:** Manuela Terranova-Barberio, Biagio Pecori, Maria Serena Roca, Serena Imbimbo, Francesca Bruzzese, Alessandra Leone, Paolo Muto, Paolo Delrio, Antonio Avallone, Alfredo Budillon, Elena Di Gennaro

**Affiliations:** 1 Experimental Pharmacology Unit, Istituto Nazionale Tumori Fondazione G. Pascale – IRCCS, Via Mariano Semmola, 13, 80131 Naples, NA Italy; 2 Radiotherapy Unit, Istituto Nazionale Tumori Fondazione G. Pascale – IRCCS, Naples, Italy; 3 Colorectal Cancer Surgery Unit, Istituto Nazionale Tumori Fondazione G. Pascale – IRCCS, Naples, Italy; 4 Abdominal Oncology Unit, Istituto Nazionale Tumori Fondazione G. Pascale – IRCCS, Naples, Italy; 5 Division of Hematology and Oncology, University of California, San Francisco, CA 94143 USA

**Keywords:** HDAC inhibitor, Valproic acid, Radiotherapy, Colorectal cancer, Capecitabine, p53

## Abstract

**Background:**

Recurrence with distant metastases has become the predominant pattern of failure in locally advanced rectal cancer (LARC), thus the integration of new antineoplastic agents into preoperative fluoropyrimidine-based chemo-radiotherapy represents a clinical challenge to implement an intensified therapeutic strategy.

The present study examined the combination of the histone deacetylase inhibitor (HDACi) valproic acid (VPA) with fluoropyrimidine-based chemo-radiotherapy on colorectal cancer (CRC) cells.

**Methods:**

HCT-116 (p53-wild type), HCT-116 p53^−/−^ (p53-null), SW620 and HT29 (p53-mutant) CRC cell lines were used to assess the antitumor interaction between VPA and capecitabine metabolite 5′-deoxy-5-fluorouridine (5′-DFUR) in combination with radiotherapy and to evaluate the role of p53 in the combination treatment. Effects on proliferation, clonogenicity and apoptosis were evaluated, along with γH2AX foci formation as an indicator for DNA damage.

**Results:**

Combined treatment with equipotent doses of VPA and 5′-DFUR resulted in synergistic effects in CRC lines expressing p53 (wild-type or mutant). In HCT-116 p53^−/−^ cells we observed antagonist effects. Radiotherapy further potentiated the antiproliferative, pro-apoptotic and DNA damage effects induced by 5′-DFUR/VPA combination in p53 expressing cells.

**Conclusions:**

These results highlighted the role of VPA as valuable candidate to be added to preoperative chemo-radiotherapy in LARC. On these bases we launched the ongoing phase I/II study of VPA and short-course radiotherapy plus capecitabine as preoperative treatment in low-moderate risk rectal cancer (V-shoRT-R3).

**Electronic supplementary material:**

The online version of this article (10.1186/s13046-017-0647-5) contains supplementary material, which is available to authorized users.

## Background

Colorectal cancer (CRC) is the third most common cancer in males and females with an estimated worldwide annual incidence of 1.3 million [1, 2] and with rectal cancer representing a 30% of its totality [3].

The management of rectal cancer varies somewhat from that of colon cancer because of the increased risk of local recurrence and a poorer overall prognosis. Preoperative fluoropyrimidine-based chemo-radiotherapy followed by surgery is the preferred treatment option for patients with stages II and III rectal disease [4, 5].

However, rectal cancer is a heterogeneous group of tumors, where different types of treatments, depending on stages and progression, are available.

Although the introduction of total mesorectal excision and preoperative radiotherapy (RT) have been revolutionary and resulted in improved local control after curative resection for rectal cancer, local relapses and distant metastasis still occur and remain a cause of recurrence worldwide [6]. This is particularly true for the “high risk” locally advanced rectal cancer (LARC) patients, also defined as the “ugly” subgroup [3]. Therefore several strategies have attempted to improve local control and reduce distant recurrence adding new cytotoxic agents into the standard treatment strategy, but this is still an ongoing challenging process [3].

Histone deacetylase inhibitors (HDACi) are an emerging group of agents that target histone deacetylase influencing chromatin structure, which in turn regulates gene expression. Radiosensitization by HDACi has been demonstrated in multiple preclinical and clinical studies [7–10]. Moreover HDACi can also modulate cellular functions independent of gene expression by acting on non-histone proteins deacetylation, in this way being involved in the regulation of different altered pathway in cancer, such as apoptosis, cell cycle and DNA repair.

Valproic acid (VPA) is an anti-epileptic drug with HDAC inhibitory activity, characterized by a much better safety profile compared to other HDACi, with neovestibular symptoms, fatigue and somnolence as the only dose-limiting toxicities [11]. VPA is also considered a less potent HDACi and this could be probably associated to its minor toxicity. For these reasons and due to its safe use as chronic therapy in epileptic disorders, VPA represents a good candidate to be tested in combination therapy development in cancer patients. A good tolerability and encouraging tumor responses of VPA in combination with chemotherapy were observed in phase I/II trials in various solid tumors, including CRC [12–16].

We have previously demonstrated that HDACi, including VPA, synergize with fluoropyrimidines, in vitro and in vivo preclinical models of breast and CRC cancer by down-regulating thymidylate synthase (TS), the key enzyme in the mechanism of action of 5-Fluorouracil (5-FU) and by up-regulating thymidine phosphorylase (TP), the key enzyme converting capecitabine to 5-FU [17–19]. TS is an essential enzyme for the de novo synthesis of thymidylate and subsequently DNA synthesis and it is a critical target for 5-FU. High levels of TS expression have been correlated with poorer overall patient survival in several tumors and resistance to 5-FU [20]. Thus, while increasing the conversion of capecitabine to 5-FU, through TP modulation, HDACi down-regulate TS, 5-FU final target, enhancing its antitumor activity. Preclinical radiosensitization activity of VPA has been also demonstrated [9, 10].

In the present study, we examined for the first time the effect of VPA in combination with fluoropyrimidines and RT on human CRC cell lines. Since p53 signaling is frequently dysregulated in CRC and the loss of a complete functional p53 is often associated with resistance to current therapies and poor prognosis, we also investigated the role of p53 in the combination setting, taking advantage of four cellular models: the HCT116 p53-wild type (wt) and its p53-null subline HCT-116 p53^−/−^, and the HT29 and SW620 p53-mutant (mut) cell lines.

## Methods

### Materials

VPA was purchased from Enzo Life Sciences (Farmingdale) while 5′-deoxy-5-fluorouridine (5′-DFUR) from Sigma-Aldrich. Stock solutions were prepared in sterile water and diluted to appropriate concentrations in culture medium before addition to the cells. All media, serum, antibiotics, and glutamine were from Lonza (Verviers).

### Cell culture and cell proliferation assay

HT29 and SW620 cell lines were from American Type Culture Collections (Rockville, MD, USA), while HCT-116 and HCT-116 p53^−/−^ were kindly provided by Prof. G. Russo (University Federico II, Naples, Italy). All cell lines were maintained in Dulbecco’s modified Eagle’s medium (DMEM) supplemented with 10% heat-inactivated foetal bovine serum, 50 units/mL penicillin, 500 μg/mL streptomycin, and 4 mmol/L glutamine. All cell lines were cultivated at 37 °C in a humidified 5% CO_2_ atmosphere, regularly inspected to be free of mycoplasma with the Mycoalert Mycoplasma Detection Kit (Lonza). Cells have been authenticated with a short tandem repeat profile generated by LGC Standards (Middlesex).

Cell survival/proliferation was performed by a spectrophotometric dye incorporation assay using sulforhodamine B (SRB, ICN Biomedicals) in quadruplicate in 96-well plates, after 96 h from treatment, as described before [17].

All in vitro studies in cancer cells were here performed with capecitabine-metabolite 5′-DFUR, which requires the presence of TP to be converted into the active 5-FU drug. Capecitabine, being a prodrug, needs a first catabolic step of conversion due to the Carboxyl esterase activity, which enzyme has low level expression in most cancer cell lines, as previously described [18].

### In vitro drugs combination studies

Drug interaction was evaluated by the Chou-Talalay method, based on concentration-effect curves generated as a plot of the fraction of unaffected (surviving) cells versus drug concentration [21, 22]. Serial dilutions of equipotent doses of the two agents in combination (VPA and 5′-DFUR) were tested. Synergism, additivity, or antagonism were quantified evaluating the combination index (CI) calculated by the Chou-Talalay equation with Calcusyn software (Biosoft) as described elsewhere [17, 23–26]. A CI < 0.9, CI = 0.9–1.2, and CI > 1.2 indicated synergistic, additive or antagonistic effect, respectively [17, 25]. The dose reduction index (DRI) determines the magnitude of dose reduction allowed for each drug when given in synergistic combination, as compared with the concentration of a single agent that is needed to achieve the same effect level [21].

### Clonogenic assay

SW620, HT29, HCT-116 and HCT-116 p53^−/−^ cells were used for colony forming assay. Briefly around 80 or 100 cells were seeded in a 6-well flat-bottom plate and treated for 24 h with VPA 1 mM and/or 5′-DFUR at a concentration corresponding at IC_15_ at 96 h. The following day, cells were placed in a water-equivalent phantom at a depth of 5 cm and exposed or not to a single 2 Gy irradiation with 6 MV photons, from an Elekta “Agility” linear accelerator. Colonies were allowed to grow for 12–14 days after RT, then collected, washed with PBS 1× and stained with 0.5% crystal violet in a solution 25% methanol in water for 30 min. Colonies were photographed, analysed and the colonies aria was evaluated using image-Pro-Plus (Immagini and Computer snc). Experiments were performed in triplicate and repeated at least 3 times.

### Immunofluorescent staining for γH2AX foci

HT29, SW620, HCT-116 and HCT-116 p53^−/−^ cells were seeded 30,000 cells/well on rounded slides placed in a 24 well flat-bottom plate. Cells were treated or not with VPA and/or 5′-DFUR at a concentration corresponding at IC_30 _or IC_50 _at 96 h for 24 h and then exposed or not to a single dose irradiation (2 Gy) as described above. Cells were then collected 24 h after RT, washed with PBS 1×, pre-fixed with formaldehyde 4% in PBS 1× for 10 min at room temperature, washed with PBS 1×, permeabilized and fixed with 100% methanol at −20 °C for 10 min. Cells were then stained for γH2AX antibody (green). After secondary antibody incubation, slides were mounted with DAPI (blue) mountant (ProLong® Gold Antifade Mountant with DAPI, Life Technologies) applied directly to fluorescently labeled cell on microscope slides. Slides were next analyzed using a fluorescence microscope (Axioscope.A1, Zeiss). Representative images show γH2AX-positive nuclear foci cells with 63× magnification.

### Protein extraction and western blotting

Cells treated as indicated were harvested, lysed and, after protein concentration was determined by Bradford method (Bio-Rad Protein Assay), separated on SDS poly-acrylamide gel electrophoresis (PAGE), as described elsewhere [17, 23]. Proteins were next transferred to nitrocellulose membranes, immunoblotted with specific antibodies and probed with the appropriate horseradish peroxidase-linked IgG. Immunoreactive bands were detected by enhanced chemiluminescence (Immobilon Western, Chemiluminescent HRP Substrate, Millipore, USA). The following primary antibodies (Abs) were used to investigate protein expression: Thymidylate Synthase (TS)-Ab (Rockand Immunochemicals Inc.; cod. 100–601-199); γ-Tubulin-Ab (cod. sc-7396), and platelet-derived endothelial growth factor (TP)-Ab (cod. sc-71,867), VDAC-1-Ab (cod. sc-8829), p53-Ab (cod. sc-6243) (Santa Cruz Biotechnology); γH2AX-Ab (cod. 05636) (Millipore); phospo-p53-Ab (cod. #9289), BAX-Ab (cod. #2774) and acetyl-H3-Ab (cod. #9649), GAPDH-Ab (cod. #2118) (Cell Signalling Technology); ATM (cod. PC116) (Calbiochem); phospho-ATM-Ab (cod. Ab81292) (Abcam). Densitometric analysis of western blotting data was performed by NIH ImageJ software.

### Flow cytometry analysis of apoptosis

HT29 and SW620 cells were treated with VPA and/or 5′-DFUR, at the indicated concentrations as described for clonogenic assay, for 24 h and then exposed or not to 2 Gy RT. Apoptosis was measured 24 and 48 h after RT using the annexin V-fluorescein isothiocyanate (annexin V-FITC). Briefly, adherent cells were harvested, washed with PBS 1× and stained with annexin V-FITC. Annexin positive cells were quantified with FACScalibur flow cytometer (Becton Dickinson), considering fluorescence collected as FL1 (Log scale) and analysed using CellQuestPro software (Becton Dickinson). Data were acquired after analysis of at least 10,000 events.

### Flow cytometry analysis of cell cycle

Analysis of cell cycle kinetic was performed at indicated times on HT29 and SW620 cells treated with VPA/5′-DFUR/RT combination treatment. Briefly, adherent and floating cells were harvested, fixed in 70% ethanol and stored at −20 °C until analysis. After nuclear DNA staining with propidium iodide, flow cytometry was evaluated by a FACScalibur flow cytometer (Becton Dickinson). For each sample, 20,000 events were collected. Cell cycle analysis was performed with ModFit LT software (Verity Software House, Inc., Topsham, ME). FL2 area versus FL2 width gating was done to exclude doublets from the G2-M region.

### Statistics

The results of in vitro cell proliferation are expressed as the means for at least three independent experiments done in quadruplicates, and the standard deviation (SD) is indicated.

Representative results from western blotting, immunofluorescent staining for γH2AX foci as well as apoptosis and cell cycle analysis by flow cytometry (perfomed in triplicates) from a single experiment are presented; additional experiments yielded similar results. Appropriate statistical analyses were applied, assuming a normal sample distribution. Statistical significance in clonogenic assay was determined by the unpaired t-test. All statistical evaluations were done using GraphPad Prism 6 (GraphPad Software, Inc.).

## Results

### In vitro synergistic antitumor effects of VPA in combination with 5′-DFUR in CRC cells: role of TP, TS and p53

**Fig. 1:**
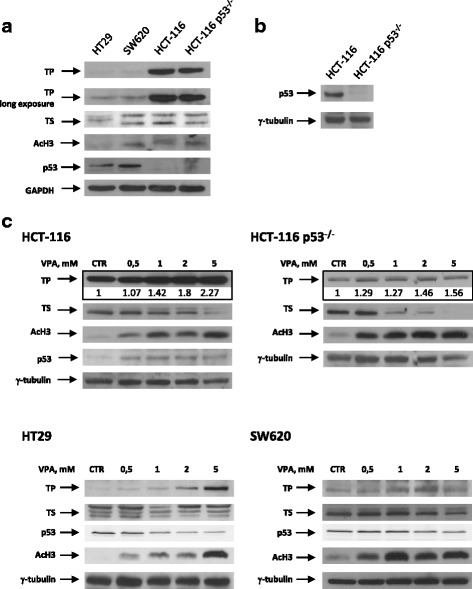
HDAC inhibitor VPA modulates TP and TS protein expression in CRC cell lines. TP, AcH3, TS (**a**) and p53 (**a** and **b**) basal protein expression was determined by western blot on the indicated CRC cells. **c** TP, TS, p53 and AcH3 protein expression was determined by western blot on HCT116, HCT116 p53^−/−^, HT29 and SW620 cells untreated or treated for 24 h with increased doses of VPA. GAPDH or γ-tubulin were used as protein loading control

We first evaluated the antiproliferative effect of either VPA or the capecitabine metabolite 5′-DFUR, as single agent, on HT29, SW620, HCT-116 and HCT-116 p53^−/−^ cell lines. All examined CRC cell lines were equally sensitive to VPA treatment, independently from their intrinsic characteristics such as p53, KRAS, BRAF, PI3KCA status (Table 1), the basal expression of TS and TP proteins, or the basal histone-H3 acetylation (AcH3) (Fig. 1a). As shown in Fig. 1a, HT29 and SW620 cells expressed lower level of TP protein compared to both HCT-116 cell lines. Moreover, being p53-mut, HT29 and SW620 cells expressed higher p53 protein levels compared to HCT-116 cells (Fig. 1a). We confirmed that HCT-116 p53^−/−^ cells did not express significant levels of p53 protein (Fig. 1b).

**Table 1:** Characteristics and sensitivity of colorectal cancer cell lines to valproic acid and 5′-deoxy-5-fluorouridine (5′-DFUR)

Cell lines	p53	KRAS	B-RAF	PI3KCA	IC50 96 h VPA, mM ± SD	IC50 96 h 5′-DFUR, μM ± SD
HT29	mut (R273H)	wt	mut	mut	3.35 ± 0.49	9.88 ± 2.14
SW620	mut (R273H)	mut (G12 V)	wt	wt	2.01 ± 0.52	35.31 ± 5.95
HCT-116	wt	mut (G13D)	wt	mut	1.41 ± 0.19	2.16 ± 0.43
HCT-116 p53^−/−^	null	mut (G13D)	wt	mut	2.35 ± 0.43	3.41 ± 0.68

IC_50_ values were computed at 96 h of treatment (mean ± standard deviation (SD) from at least three separate experiments performed in quadruplicates); (mut is for mutant and wt for wild-type)

Interestingly, in the two p53-mut HT29 and SW620 cells, the lower expression of TP, the critical enzyme converting 5′-DFUR into the active compound 5-FU, correlates with the lower sensitivity to 5′-DFUR (Table 1 and Fig. 1a), consistently with our previous studies [18, 19].

We next investigated VPA antitumor effect in combination with 5′-DFUR. Combined treatment with equipotent doses (50:50 cytotoxic ratio) of VPA and 5′-DFUR for 96 h, resulted in synergistic antiproliferative effect in HT29, SW620 and HCT-116 cell lines, as shown by CI values always lower than 0.9, calculated at 50% (CI_50_), 75% (CI_75_) or 90% (CI_90_) of cell lethality (Table 2). In addition, we demonstrated a reduction in the IC_50_ values (DRI_50_) for both VPA and 5′-DFUR in the combination setting compared with the two drugs used alone (Table 2). Interestingly, in HCT-116 p53^−/−^we did not observe synergistic interaction between VPA and 5′-DFUR, as shown by the CI values higher than 1.2 and the lower DRI for 5′-DFUR compared with the HCT-116 p53-wt cells (Table 2). Similar data were obtained in p53-wt, -mut and -null prostate cancer and non-small-cell lung cancer models. Here, we demonstrated a synergistic interaction between VPA/5′-DFUR in p53-wt or p53-mut cell lines, but not in p53-null cells, where we observed only an additive/antagonistic effect (unpublished results).

**Table 2:** Combination index (CI) and dose reduction index (DRI) values for VPA and 5′-DFUR combination treatment

Cell lines	^a^CI_50_ ± SD VPA + 5′-DFUR	^a^CI_75_ ± SD VPA + 5′-DFUR	^a^CI_90_ ± SD VPA + 5′-DFUR	^b^DRI at IC_50_ ± SD VPA 5′-DFUR
HT29	0.87 ± 0.17	0.75 ± 0.07	0.75 ± 0.078	1.65 ± 0.35 2.56 ± 0.62
SW620	0.81 ± 0.10	0.83 ± 0.16	0.74 ± 0.24	2.91 ± 1.49 1.90 ± 0.40
HCT-116	0.87 ± 0.02	0.86 ± 0.04	0.77 ± 0.03	1.85 ± 0.28 2.44 ± 0.22
HCT-116 p53^−/−^	1.24 ± 0.15	1.15 ± 0.03	1.15 ± 0.17	1.43 ± 0.24 1.75 ± 0.33

^a^CI values (mean ± SD from at least three separate experiments performed in quadruplicates) computed at 50, 75 and 90% of cell kill (CI_50_, CI_75_and CI_90_, respectively) according by CalcuSyn software after 96 h of treatment. Combinations were considered strongly synergistic when CIs were below 0.9. ^b^DRI values (mean ± SD . from at least three separate experiments performed in quadruplicates) represents the order of magnitude (fold) of dose reduction obtained for IC_50_ (DRI_50_) in combination setting compared with each drug alone

To gain insight into the mechanism of the observed synergism, we tested the effect of increasing doses of VPA on TS, TP and p53 protein expression in HCT116 p53-wt and HCT116 p53^−/−^ cells and in p53-mut HT29 and SW620 cells. VPA, even at low doses (such as 0.5 and 1 mM), was able to up-regulate TP and to down-regulate TS protein expression, within 24 h of treatment in all cell lines examined, in a dose-dependent manner, independently of p53 expression and status, as previously reported with alternative HDACi and/or other cell models [17] (Fig. 1c). Moreover, we observed that VPA up-regulates p53-wt and down-regulates p53-mut protein levels, in accord with previous data obtained by our group and others, using alternative HDACi [17, 27]. Induction of AcH3 confirmed the dose-dependent HDAC-inhibitory activity of VPA in all treated cells (Fig. 1c).

### VPA/5′-DFUR combination sensitizes CRC cells to RT: role of p53

To evaluate if VPA/5′-DFUR combination can sensitize CRC cells to RT, cells were first treated for 24 h with VPA and/or 5′-DFUR and next exposed or not to 2 Gy RT. Notably, we performed most of further experiments with a low dosage of VPA (1 mM), easily reached in the plasma of patients treated with antiepileptic dosage [28] and also able to modulate TS and TP expression (Fig. 1c).

**Fig. 2:**
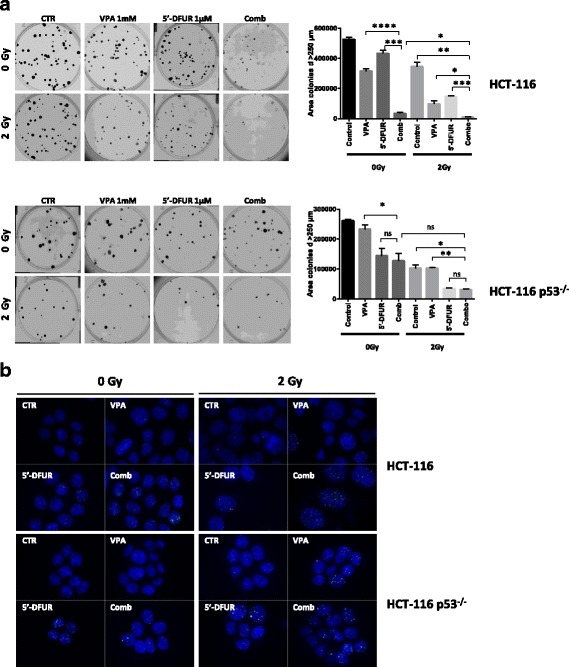
Crucial role of p53 in the synergistic effect of VPA/5′-DFUR combination treatment with RT. **a** Clonogenic assay shows the long-term effects of combination treatment VPA/5′-DFUR plus 2 Gy RT on CRC cell lines in HCT-116 and HCT-116 p53^−/−^ collected 12–14 days after RT. A photograph of one well in a representative experiment is shown for each treatment; bar graphs show the area of colony with diameter > 250 μm (mean ± SD of 2 or more separate experiments each one with technical triplicate). * = *p* < 0.05; ** = *p* < 0.006; *** = *p* < 0.0005. **b** DNA damage was analyzed in HCT-116 and HCT-116 p53^−/−^ cell lines by visualizing DSB marker γH2AX foci. Cells treated for 24 h with or without VPA and/or 5′-DFUR at the indicated concentration, corresponding to IC_30_ at 96 h and then with or without 2 Gy RT, were collected 24 h after RT. Cells were fixed, stained for γH2AX (green) and DAPI for nuclei (blue) and observed by microscope. Representative images show γH2AX-positive nuclear foci cells with 63× magnification

We used colony formation assay and initially evaluated VPA/5′-DFUR plus RT on HCT-116 and HCT-116 p53^−/−^cells. As shown in Fig. 2a, VPA/5′-DFUR treatment strongly reduced colony formation in HCT-116 cells compared to control or single agent treatments. However, this effect was not observed in the syngeneic HCT-116 p53^−/−^cell line, confirming the data reported above by antiproliferative assay and CI evaluation. Furthermore, although synergistic inhibitory effect was observed by combining either VPA or 5′-DFUR with RT, VPA/5′-DFUR plus RT triple combination almost completely inhibited colony formation of HCT116 cells. HCT-116 p53^−/−^ cells appeared more sensitive to either RT or 5′-DFUR alone compared to parental cells, and the antitumor effect of RT was further increased in combination with 5′-DFUR. However in this cell line we did not observe any synergistic effect of RT in combination with VPA alone or in triple combination, being 5′-DFUR single agent treatment, with or without RT, comparable to VPA/5′-DFUR combined treatments (Fig. 2a).

We also evaluated the treatments effect on DNA damage, by measuring γH2AX foci formation 24 h after RT. As shown in Fig. 2b, in HCT-116 cells VPA/5′-DFUR treatment increased the number of γH2AX foci compared to single agent treatments and this effect was clearly amplified by RT (Fig. 2b and Additional file 1: Figure S1A). Conversely, in agreement with colony formation experiments, in the HCT-116 p53^−/−^ cells, although RT alone appeared more effective in increasing γH2AX foci formation compared to untreated cells, VPA and 5′-DFUR in combination did not demonstrate any synergistic effect, neither alone nor with RT, compared to single agent treatments (Fig. 2a and Additional file 1: Figure S1B).

**Fig. 3:**
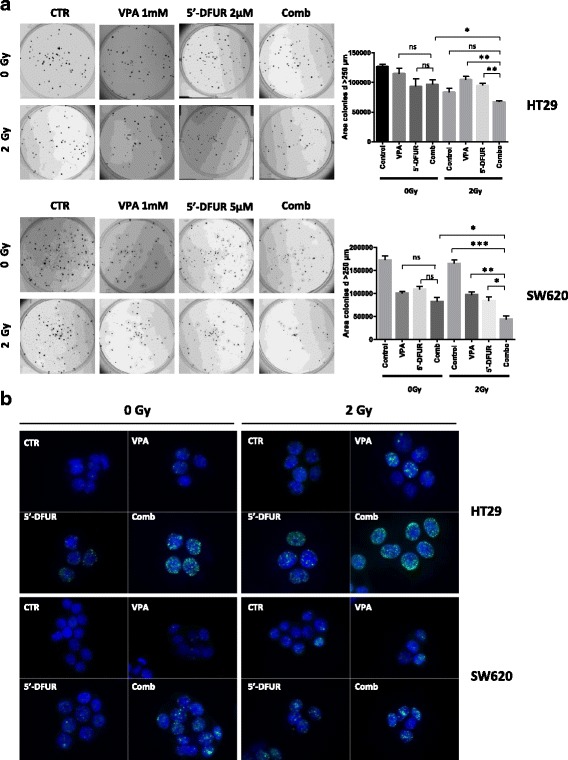
Synergistic antiproliferative effect induced by VPA/5′-DFUR combination plus 2 Gy RT in CRC cell lines. HT29 and SW620 cells were treated or untreated with VPA 1 mM and 5′-DFUR at the indicated concentrations, corresponding to IC_30_ at 96 h for 24 h and then with or without 2 Gy RT; **a** Clonogenic assay shows the long-term effects of combination treatment VPA/5′-DFUR plus 2 Gy RT on CRC cell lines HT29 and SW620 collected 12–14 days after RT. A photograph of one well in a representative experiment is shown for each treatment; bar graphs show the area of colony with diameter > 250 μm (mean ± SD of 2 or more separate experiments each one with technical triplicate). * = *p* < 0.05; ** = *p* < 0.006; *** = *p* < 0.0005. **b** DNA damage was analyzed in HT29 and SW620 by visualizing DSB marker γH2AX foci. Cells treated for 24 h with or without VPA and/or 5′-DFUR at the indicated concentrations, corresponding to IC_30_ at 96 h and then with or without 2 Gy RT, were collected 24 h after RT. Cells were fixed, stained for γH2AX (green) and DAPI for nuclei (blue) and observed by microscope. Representative images show γH2AX-positive nuclear foci cells with 63× magnification

We next tested VPA/5′-DFUR and RT interaction in HT29 and SW620 p53-mut cell lines. As shown by colony formation assay, VPA/5′-DFUR combination did not significantly increase the antitumor effect compared to single agent treatments in both cell lines. Similarly, the addition of RT to single agent treatments did not improve the antitumor effect in p53-mut cells. Conversely, a significant inhibition of colony formation was observed only in triple combination setting in both HT29 and SW620 cells (Fig. 3a).

Furthermore, although we did not observe any synergistic effect in VPA/5′-DFUR combination by colony formation assay, we showed an increased number of γH2AX foci after VPA/5′-DFUR treatment compared to control or single drugs, in both HT29 and SW620 cell lines (Fig. 3b). The addition of VPA/5′-DFUR to RT was able to significantly increase γH2AX foci formation in the two cell lines (Fig. 3b and Additional file 2: Figure S2A and B).

**Fig. 4:**
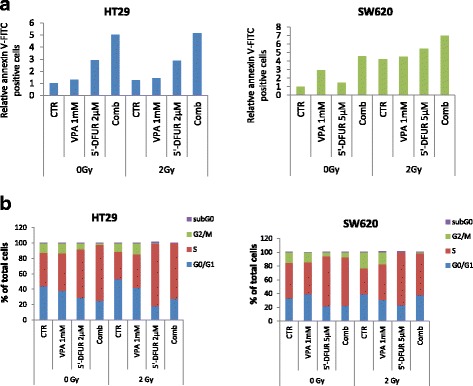
Pro-apoptotic effect induced by VPA/5′-DFUR combination plus 2 Gy RT in CRC cell lines. HT29 and SW620 cells were treated or untreated with VPA 1 mM and 5′-DFUR at the indicated concentration, corresponding to IC_30 _at 96 h for 24 h followed or not by 2 Gy RT. **a** Apoptotic effect on HT29 and SW620 cells was evaluated by flow cytometry analysis upon Annexin V-FITC staining after treatment with or without VPA 1 mM and/or 5′-DFUR at the indicated concentration, corresponding to IC_15_ at 96 h for 24 h and then with or without 2 Gy RT for 48 (HT29) and 24 (SW620) hours. **b** Cell cycle analysis was performed in HT29 and SW620 cell lines. The percentage of sub G1, G1, S and G2/M population were analyzed by flow cytometry on cells treated for 24 h with or without VPA 1 mM and 5′-DFUR at the indicated concentrations, corresponding to IC_15_ at 96 h, followed or not by 24 h exposure to 2 Gy RT

Apoptotic bodies were observed in both HT29 and SW620 cells after 5′-DFUR or VPA/5′-DFUR combination treatment, as shown by phase-contrast microscopy, and this effect was further potentiated when RT was added, particularly in VPA/5′-DFUR/RT triple combinations (Additional file 3: Figure S3). Similarly, in SW620 cells we observed an induction of apoptosis upon VPA/5′-DFUR combination treatment compared to single agent treatments, further potentiated by 24 h exposure to RT, as demonstrated by flow cytometry analysis with annexin V-FITC staining. In RT-resistant HT29 cells no major induction of apoptosis was observed after 48 h of RT treatment, compared to cells not exposed to RT either alone or in combination (Fig. 4a).

Thereafter we analyzed the effects of our treatments on cell cycle modulation (Fig. 4b). It has been reported [29] that RT induced brief and moderate G2/M arrest with a peak at 6 h and returning to basal level at 10 to 12 h following 2 Gy RT. We observed a slight increase of G2/M arrest in SW620 cells after 24 h of treatment (15.6% of cells in G2/M phase in control cells vs 23.2% in 2 Gy RT irradiated cells), but not in RT resistant HT29 cells (11.41% *vs *12.3%). Moreover we confirm in SW620 cells the ability of HDACi to abrogate the G2 arrest induced by RT as previously reported [29] (Fig. 4b).

Additionally, we observed a strong S-phase arrest after 5′-DFUR treatment in both HT29 and SW620 and this was further increased by VPA in HT29 cells. In both cell lines RT reduce S-phase arrest compared to 5′-DFUR treated cells (Fig. 4b).

In order to better define the mechanism of VPA/5′-DFUR and RT interaction in p53-mut cells, we next evaluated the expression of critical proteins potentially involved in the observed antitumor effects in p53-mut HT29 and SW620 cells by western blotting. Cells were treated for 24 h with VPA and/or 5′-DFUR, then exposed or not to 2 Gy RT and harvested after 48 h.

**Fig. 5:**
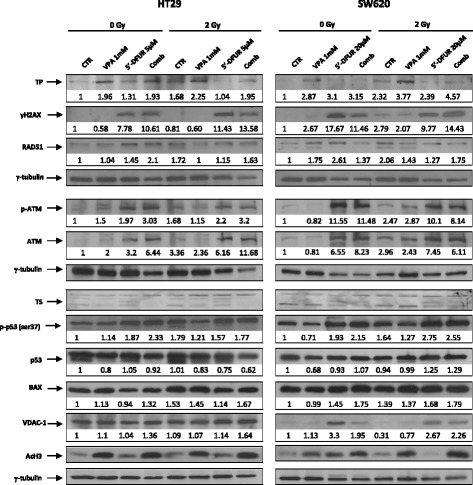
Effect of triple combination on the expression and activity of crucial proteins in DNA damage/repair pathways. HT29 and SW620 cells were treated or untreated with VPA 1 mM and 5′-DFUR at the indicated concentrations, corresponding to IC_30 _at 96 h for 24 h followed or not by 2 Gy RT. TP, TS, γH2AX, phospho-ATM, ATM, phosphop53, p53, BAX, VDAC-1, and AcH3 protein expression were evaluated by western blot analysis 48 h after RT. γ-tubulin was used as protein loading control

First of all we confirmed that VPA alone or in combination with 5′-DFUR, in the presence or absence of RT is able to increase TP protein levels also at a later time point (after 72 h). After 48 h, RT alone slightly increased TP in both cell lines, in accord with previous reports [30, 31]. This effect is further potentiated by VPA. Notably, as confirmed by densitometric analysis, TP up-regulation is conserved (HT29 cells) or even potentiated (SW620 cells) in triple combination setting (Fig. 5). Moreover, we confirm that VPA reduces both basal and 5′-DFUR-induced TS protein expression as previously reported [17–19], in the presence or absence of RT. Notably, the formation of the ternary complex between the 5-FU metabolite FdUMP, the enzyme TS and 5,10-methylene tetrahydrofolate [17], highlighted by the upper bands in the western blot, is still achieved in the presence of VPA, indicating that TS downregulation does not affect the biochemical inhibition of the enzyme induced by 5-FU.

We also confirmed that VPA/5′-DFUR combination treatment was able to induce DNA damage as demonstrated by γH2AX increased protein expression. Significantly the triple combination setting further increased γH2AX foci formation, in agreement with previous results (Fig. 5). Notably, we also demonstrated a prolonged DNA damage up to 48 h after RT in combination setting.

In agreement with these latter findings, upon VPA/5′-DFUR treatment with RT, we observed, in both HT29 and SW620 cell lines, a prolonged induction in the phosphorylation of ATM, the kinase mainly recognizing DNA-double-strand breaks (DSB) by ionizing radiation, compared with RT alone. In both cell lines ATM phosphorylation was strongly induced also by VPA/5′-DFUR combination and in SW620 cells also by 5′-DFUR treatment alone (Fig. 5). The observed ATM phosphorylation/activity increase correlates with an increase in ATM protein expression.

Indeed, we also observed an induction of p53 phosphorylation (serine 37) in both cell lines probably mediated by ATM induction, in combination treatment. In details, this is evident in the absence of RT, while p53 phosphorylation induction in triple combination is similar to that observed in 5′-DFUR/RT treated cells.

Furthermore, in both HT29 and SW620 cells, the p53 activation induced upon VPA/5′-DFUR/RT combination treatment, is accompanied by the induction of the pro-apoptotic protein BAX, as compared to control or single agent treatments, an observation that can explain, at least in part, the apoptotic induction (Fig. 4a).

Finally, we evaluated the expression of voltage-dependent anion-selective channel protein 1 (VDAC-1), a protein involved in reactive oxygen species (ROS) generation and a key player in mitochondria-mediated apoptosis, that we have previously reported to be regulated by HDACi [32]. As shown in Fig. 5 in HT29 cells, we observed an increased expression of VDAC- 1 after VPA/5′-DFUR treatment compared to single agent alone, further enhanced by RT. In SW620, in the absence of RT, we observed a clear increase in VDAC-1 expression after 5′-DFUR treatment alone. This effect was maintained in the presence of RT, also in the triple combination treatment.

## Discussion

Fluoropyrimidine-based chemo-radiotherapy is a standard preoperative approach in LARC patients. HDACi have shown promising anticancer effects in both preclinical and clinical setting as radiosensitizers when administrated in combination with RT [7, 8, 33–35].

In this study we report that the HDACi VPA in combination with capecitabine could be a suitable approach to use in combination with RT in CRC treatment in both p53-wt and p53-mut tumors.

We and others have previously demonstrate that HDACi, including VPA, synergize with either 5-FU or capecitabine because are able to modulate the levels of two critical enzymes in the metabolism of fluoropyrimidines such as TP and TS [17, 18, 36–39]. Notably, TP knockdown experiments confirmed a crucial role of TP protein up-regulation in the observed synergism [19]. In the present study we discovered for the first time that VPA/capecitabine combination treatment further synergizes with RT, as previously reported with the pan-HDACi vorinostat [35]. Moreover, we also confirmed modulation of both TS and TP protein levels by VPA in CRC models, even in the presence of RT. Interestingly, TP protein induction is achieved also at low doses of VPA (0.5–1 mM), corresponding to a plasma level between 50 and 100 μg/ml, easily reached in patients with normal anticonvulsant doses [28]. Although at these doses VPA did not induce growth inhibition as single agents, a significant synergistic antitumor effect was still demonstrated in combination with 5′-DFUR and RT, suggesting a specific mechanism of interaction as well as the feasibility to translate this approach in a clinical study.

Furthermore, although our data suggest that VPA may increase sensitivity to fluoropyrimidines by specifically modulating both TS and TP expression, we also showed that p53 has a critical role in the observed synergism. Indeed, although we demonstrated that in p53-null HCT-116 p53^−/−^cells VPA still modulates both TS and TP, no synergistic antitumor effect was observed in combination with 5′-DFUR and/or RT in this cell lines compared with p53-wt or p53-mut cells. Notably, we confirmed similar results in other cancer models. HDACi, such as VPA and vorinostat, are able to induce apoptosis independently of p53 status, while for others, such as entinostat, p53 is crucial for their activity [40, 41]. However, previous reports have demonstrated that HDACi radiosensitization is p53-influenced through p53 acetylation-mediated c-myc down regulation [27]. We and others have shown that HDACi might restore/induce p53-wt expression by modulating the epigenetic suppression of the gene or by inhibiting protein degradation [17, 41, 42], in this way being able to potentiate anticancer drugs effect, promoting apoptosis. HDACi are also able to downregulate mutated p53, by transcriptional mechanism or by accelerating mutant protein degradation [17, 43, 44], in this way abrogating gain of function oncogenic properties, including mechanism of resistance to anticancer drugs. On the contrary, we speculate that, when p53 is deleted, cancer cells rely on different mechanisms in order to acquire resistance to anticancer drugs and, thus, the synergistic p53-dependent effect exerted by HDACi in combination with anticancer drugs is lost.

HDACs have recently been found to participate in the DNA damage response and their down-regulation has been associated with impaired DNA repair [45]. Considering that after 24 h the RT-induced γH2AX foci formation, and indicator of DSB, should have recovered to control levels, our data demonstrating a prolonged DNA damage up to 48 h after RT in combination setting, suggest that, mechanistically, VPA was able to prolong and further increase the DNA damage induced by 5′-DFUR and/or RT. This effect, most likely due to a decrease of repair rate of DSB, results in apoptosis and in the potentiation of the antitumor effect, specifically in the triple combination. As expected, this effect is particularly evident in p53-wt cell lines, but was abolished in p53-null cells. Remarkably, VPA/5′-DFUR/RT triple synergistic prolongation of DNA damage was also demonstrated in p53-mut cell lines.

Accordingly, we demonstrated in p53-mut HT29 and SW620 cells that VPA in combination with 5′-DFUR and RT increased p53 phosphorylation at serine 37, a site of phosphorylation identified in cells following DNA damage after exposure to radiation [46]. It was reported that in both HT29 and SW620, despite mutated, p53 can be activated by phosphorylation and can modulate cell growth or death [47–49].

In response to DSB and DNA damage, ATM recognizes it and activates Chk2 that, subsequently, by phosphorylation activates p53 [50]. In details, in the context of p53-wt, stabilization and activation of p53 induces long-term cell-cycle arrest, apoptosis, or senescence by transcriptionally regulating, among others, the CDK inhibitor p21 and the pro-apoptotic protein BAX [51]. These effects do not count in the presence of p53-mut, where p21 is not expressed and apoptosis events follow different pathways [52].

Generally, ATM phosphorylation is an early event in DNA damage [25]. Hehlgans et al. observed an induction of pATM upon 6 Gy treatment alone or in combination with a novel HDACi (NDACI054) after 0.25 h with a peak after 1 h. The ATM phosphorylation returns to basal levels 24 h after RT treatment [34]. We observed an increase in ATM phosphorylation up to 48 h post RT treatment. This prolonged effect was probably due to an increased in ATM protein expression in both HT29 and SW620 cell lines. Thus, we hypothesized that tumor cells induce ATM protein expression in order to maintain its activity as a consequence of the prolonged damage induced in combination setting and the inability to repair it.

The ATM protein is a crucial player for the induction of cell cycle arrest following DSB generation, through cell cycle checkpoints (G1, intra S and G2/M). This phenomenon leads to efficient repair of DSB or cell death [53, 54]. It has been reported that the abrogation of radiation-induced G2-M arrest by HDACi may decrease the time available for repair of DNA damage or may interfere with repair mechanisms [55], driving cells in apoptosis [56]. We observed only minor effects on cell cycle regulation in HT29 and SW620 cells, probably due to the presence of a mutated form of p53 in these cells or to the timing chosen in the analysis. Indeed Kim et al. [29] showed that the moderate G2/M arrest induced by RT was brief, with a peak at 6 h, and returning to basal level 10/12 h following 2 Gy RT. We observed only a slight increase of cells in G2/M in SW620 cells after 24 h of RT treatment, but not in RT resistant HT29 cells. It is important to underline that the strong S-phase arrest after 5′-DFUR treatment in both HT29 and SW620 was reduced in the triple combination treatment. This suggests that this mechanism could contribute to avoid DNA repair and prevent cell cycle progression.

Furthermore, we observed an induction of apoptosis upon VPA/5′-DFUR combination treatment compared to single agent treatments in both p53-mut HT29 and SW620 cell lines. This effect is further potentiated by 24 h exposure to RT in SW620 cells, but not in HT29 RT resistant cells. We have previously demonstrated that the pro-apoptotic effect induced by the synergistic combination between HDACi vorinostat and EGFR inhibitors, was mediated by an altered mitochondria homeostasis, resulting in ROS accumulation [32]. We also found that vorinostat induced the expression of VDAC-1, the major mitochondrial porin of the outer mitochondrial membrane, involved in ROS generation and key player in mitochondria-mediated apoptosis. In its open state, VDAC-1 induces apoptosis mediating the translocation of the pro-apoptotic protein BAX, the release of cytochrome c and the activation of caspases [32, 57]. Thus, VDAC-1 regulation could be functionally involved in oxidative-stress-dependent-apoptosis. In the current study our observations also suggest a possible role of VDAC-1 in the pro-apoptotic effect of VPA/5’-DFUR/RT combination in p53-mut cells. The concomitant increase in BAX protein could be the mechanism through which the triple combination treatment increases apoptosis. Indeed, it was reported that BAX may physically interact with VDAC-1 to yield a heterocomplex with increased permeability compared to VDAC-1 oligomer alone. This increases both ROS and cytochrome c release into the cytoplasm and activates apoptosis [58, 59].

Taken together, our data suggested that the synergistic interaction between VPA, 5′-DFUR and RT can results in a convergent mechanism that induce impaired regulation of DNA repair pathway targeting ATM and the downstream partners, together with an alteration in ROS accumulation that leads to DNA damage and apoptosis. Remarkably, our results showed that this combination could be used even in the more complicated and poorly prognosis-characterized subset of p53-mutated patients, casting a new light on this approach.

## Conclusion

In conclusion our findings show that the addition of VPA/capecitabine to RT is a feasible and promising strategy to improve the efficacy of preoperative treatment of LARC. On these bases we launched a phase I/II clinical study (V-ShoRT-R3 trial) [60] to explore whether the addition of both VPA and capecitabine to short-course RT before optimal radical surgery, might increase the pathologic complete tumor regression rate in low-moderate risk rectal cancer patients (ClinicalTrials.gov number NCT01898104). Correlative studies, comparing normal mucosa with tumor and on blood samples, could identify predictive biomarkers and could add new insight into the mechanism of interaction between VPA, capecitabine and RT. In this regard, the impact of p53-null type will be explored because it may give a clue to a subset of patients that could not respond to the combinatory regimen.

## Additional files

Additional file 1: Figure S1. DNA damage was analyzed in HCT-116 (**A**) and HCT-116 p53^−/−^ (**B**) by visualizing DSB marker γH2AX foci. Cells were treated with or without VPA and/or 5′-DFUR for 24 h at the indicated concentration: 1 and 1.5 mM for VPA corresponding to IC_30_ at 96 h for HCT-116 and HCT-116 p53^−/−^ respectively; 1 μM for 5′-DFUR corresponding to IC_30_ for both cell lines and 2 and 5 μM for 5′-DFUR corresponding to and IC_50_ for HCT-116 and HCT-116 p53^−/−^ respectively at 96 h. Cells were then exposed or not to 2 Gy RT and then collected 24 h after RT, fixed and stained for γH2AX (green) and DAPI for nuclei (blue) and observed by microscope. Triplicates images of a representative experiment show γH2AX-positive nuclear foci cells with 63× magnification. (PPT 3021 kb)

Additional file 2: Figure S2. DNA damage was analyzed in HT29 and SW620 by visualizing double strand break marker γH2AX foci. Cells were treated for 24 h with or without VPA and/or 5′-DFUR at the indicated concentration, corresponding to IC_30_ for VPA and IC_30_ and IC_50_ for 5′-DFUR at 96 h. Cells were then exposed or not to 2 Gy RT and then collected 24 h after RT, fixed and stained for γH2AX (green) and DAPI for nuclei (blue) and observed by microscope. Triplicates images of a representative experiment show γH2AX-positive nuclear foci cells with 63× magnification. (PPT 2793 kb)

Additional file 3: Figure S3. HT29 and SW620 cells were treated or untreated with VPA 1 mM and 5′-DFUR at the indicated concentration, corresponding to IC_30_ at 96 h for 24 h followed or not by 2 Gy RT. Morphology and survival were examined after 48 h and phase contrast images of representative area are showed. A photograph of one well in a representative experiment is shown for each treatment. (PPT 6438 kb)

## Abbreviations

5′-DFUR: 5′-deoxy-5-fluorouridine
5-FU: 5-Fluorouracil
Abs: antibodies
AcH3: acetyl-histone H3
CI: combination index
CRC: colorectal cancer
DRI: dose reduction index
DSB: DNA-double-strand breaks
FITC: fluorescein isothiocyanate
HDACi: histone deacetylase inhibitor
LARC: locally advanced rectal cancer
mut: mutant
ROS: reactive oxygen species
RT: radiotherapy
SD: standard deviation
SRB: sulforhodamine B
TP: thymidine phosphorylase
TS: thymidylate synthase
VDAC-1: voltage-dependent anion-selective channel protein 1
VPA: valproic acid
wt: wild type
