# Supplementary material for: Synergistic antitumor interaction between valproic acid, capecitabine and radiotherapy in colorectal cancer: critical role of p53
Source: J Exp Clin Cancer Res. 2017 Dec 6;36:177. doi: 10.1186/s13046-017-0647-5 (PMC5719792; doi:10.1186/s13046-017-0647-5)
Supplement: Supplementary file 1 — DNA damage was analyzed in HCT-116 (A) and HCT-116 p53−/− (B) by visualizing DSB marker γH2AX foci. Cells were treated with or without VPA and/or 5′-DFUR for 24 h at the indicated concentration: 1 and 1.5 mM for VPA corresponding to IC30 at 96 h for HCT-116 and HCT-116 p53−/− respectively; 1 μM for 5′-DFUR corresponding to IC30 for both cell lines and 2 and 5 μM for 5′-DFUR corresponding to and IC50 for HCT-116 and HCT-116 p53−/− respectively at 96 h. Cells were then exposed or not to 2 Gy RT and then collected 24 h after RT, fixed and stained for γH2AX (green) and DAPI for nuclei (blue) and observed by microscope. Triplicates images of a representative experiment show γH2AX-positive nuclear foci cells with 63× magnification. (PPT 3021 kb) [file 13046_2017_647_MOESM1_ESM.ppt]

## Slide 1
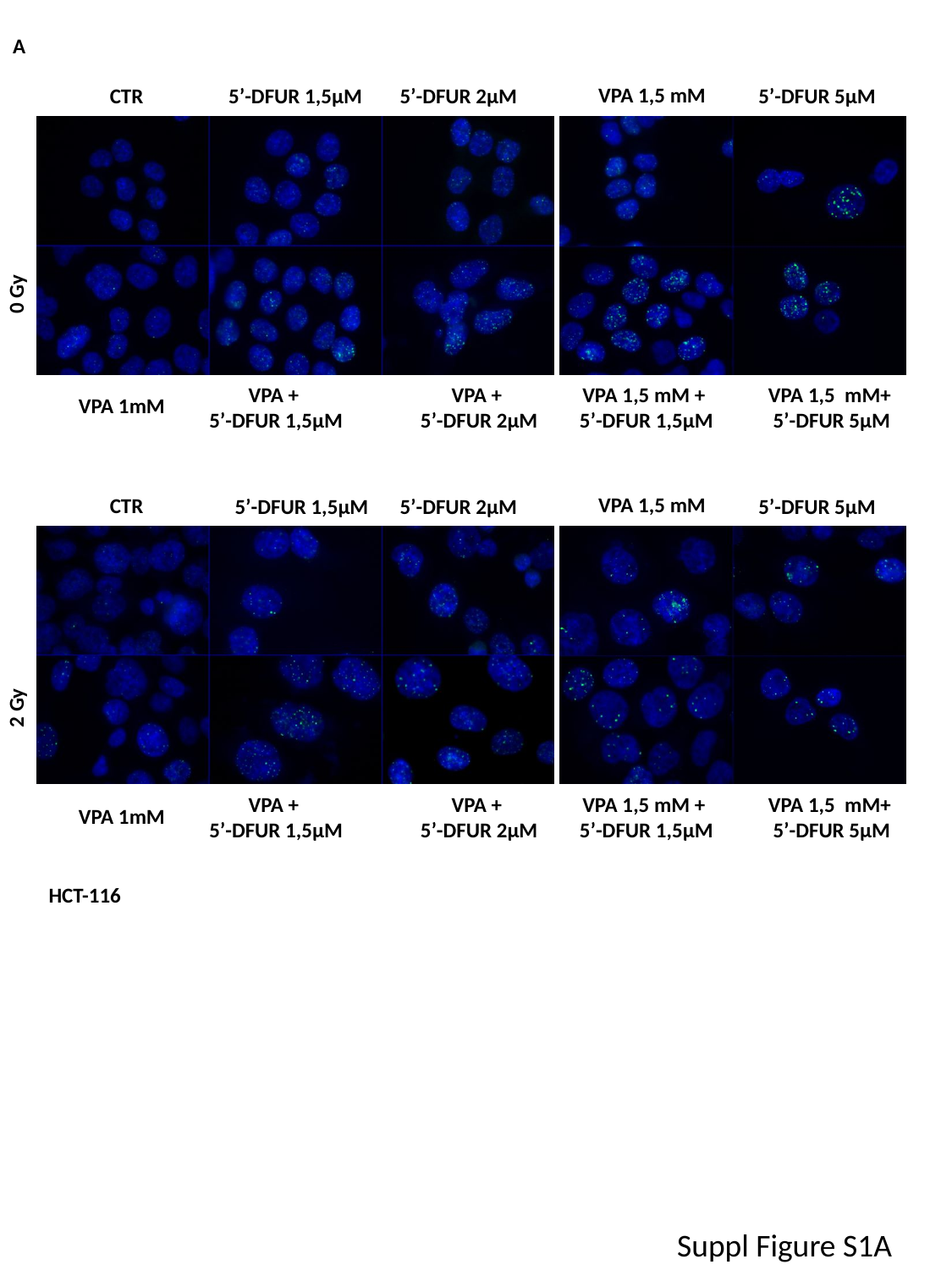

A
VPA 1,5 mM
CTR
5’-DFUR 1,5µM
5’-DFUR 2µM
5’-DFUR 5µM
0 Gy
VPA +
5’-DFUR 1,5µM
VPA +
5’-DFUR 2µM
VPA 1,5 mM +
5’-DFUR 1,5µM
VPA 1,5 mM+
 5’-DFUR 5µM
VPA 1mM
VPA 1,5 mM
CTR
5’-DFUR 1,5µM
5’-DFUR 2µM
5’-DFUR 5µM
2 Gy
VPA +
5’-DFUR 1,5µM
VPA +
5’-DFUR 2µM
VPA 1,5 mM +
5’-DFUR 1,5µM
VPA 1,5 mM+
 5’-DFUR 5µM
VPA 1mM
HCT-116
Suppl Figure S1A

## Slide 2
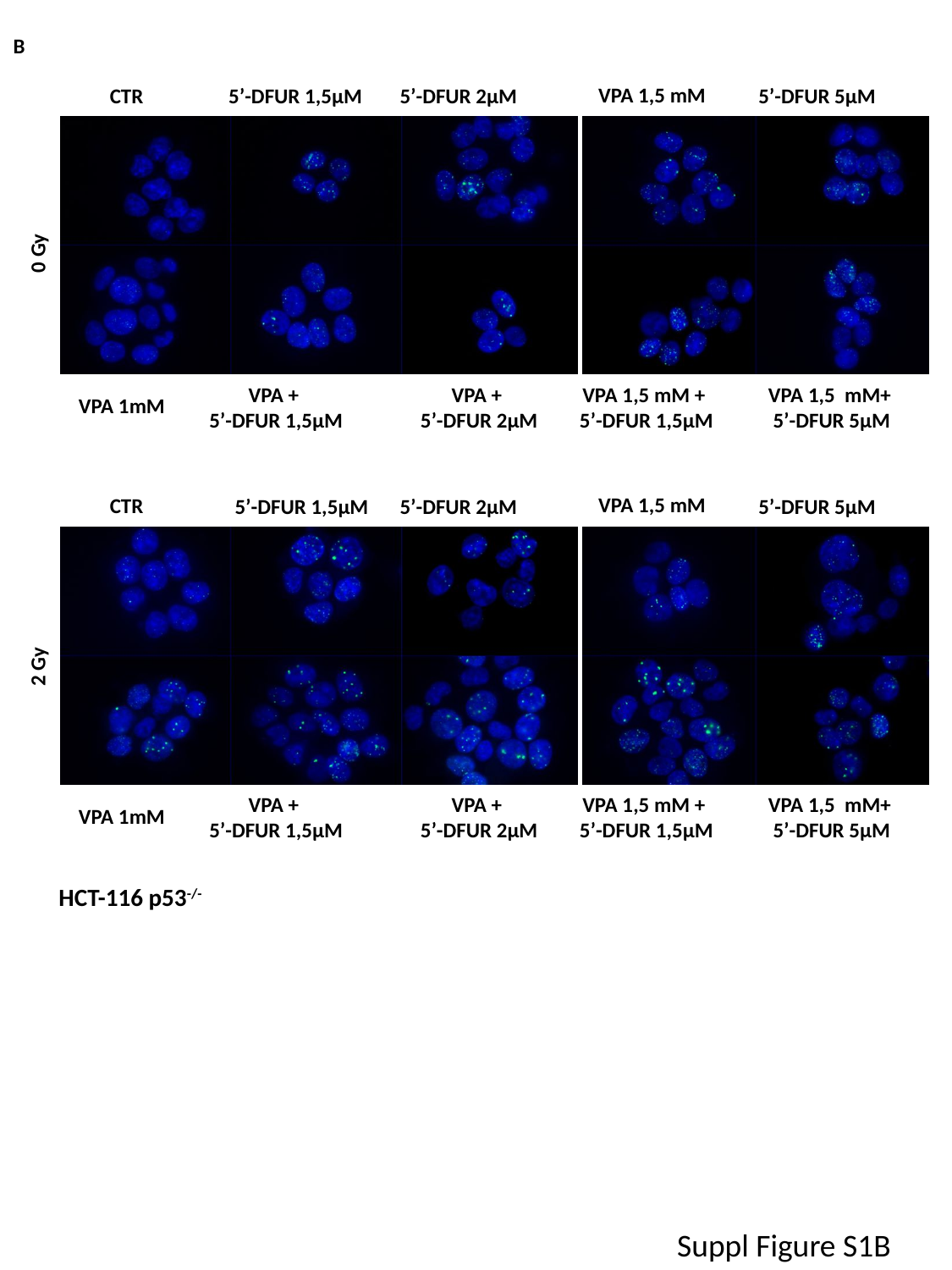

B
VPA 1,5 mM
CTR
5’-DFUR 1,5µM
5’-DFUR 2µM
5’-DFUR 5µM
0 Gy
VPA +
5’-DFUR 1,5µM
VPA +
5’-DFUR 2µM
VPA 1,5 mM +
5’-DFUR 1,5µM
VPA 1,5 mM+
 5’-DFUR 5µM
VPA 1mM
VPA 1,5 mM
CTR
5’-DFUR 1,5µM
5’-DFUR 2µM
5’-DFUR 5µM
2 Gy
VPA +
5’-DFUR 1,5µM
VPA +
5’-DFUR 2µM
VPA 1,5 mM +
5’-DFUR 1,5µM
VPA 1,5 mM+
 5’-DFUR 5µM
VPA 1mM
HCT-116 p53-/-
Suppl Figure S1B
