# Supplementary material for: Synergistic antitumor interaction between valproic acid, capecitabine and radiotherapy in colorectal cancer: critical role of p53
Source: J Exp Clin Cancer Res. 2017 Dec 6;36:177. doi: 10.1186/s13046-017-0647-5 (PMC5719792; doi:10.1186/s13046-017-0647-5)
Supplement: Supplementary file 2 — DNA damage was analyzed in HT29 and SW620 by visualizing double strand break marker γH2AX foci. Cells were treated for 24 h with or without VPA and/or 5′-DFUR at the indicated concentration, corresponding to IC30 for VPA and IC30 and IC50 for 5′-DFUR at 96 h. Cells were then exposed or not to 2 Gy RT and then collected 24 h after RT, fixed and stained for γH2AX (green) and DAPI for nuclei (blue) and observed by microscope. Triplicates images of a representative experiment show γH2AX-positive nuclear foci cells with 63× magnification. (PPT 2793 kb) [file 13046_2017_647_MOESM2_ESM.ppt]

## Slide 1
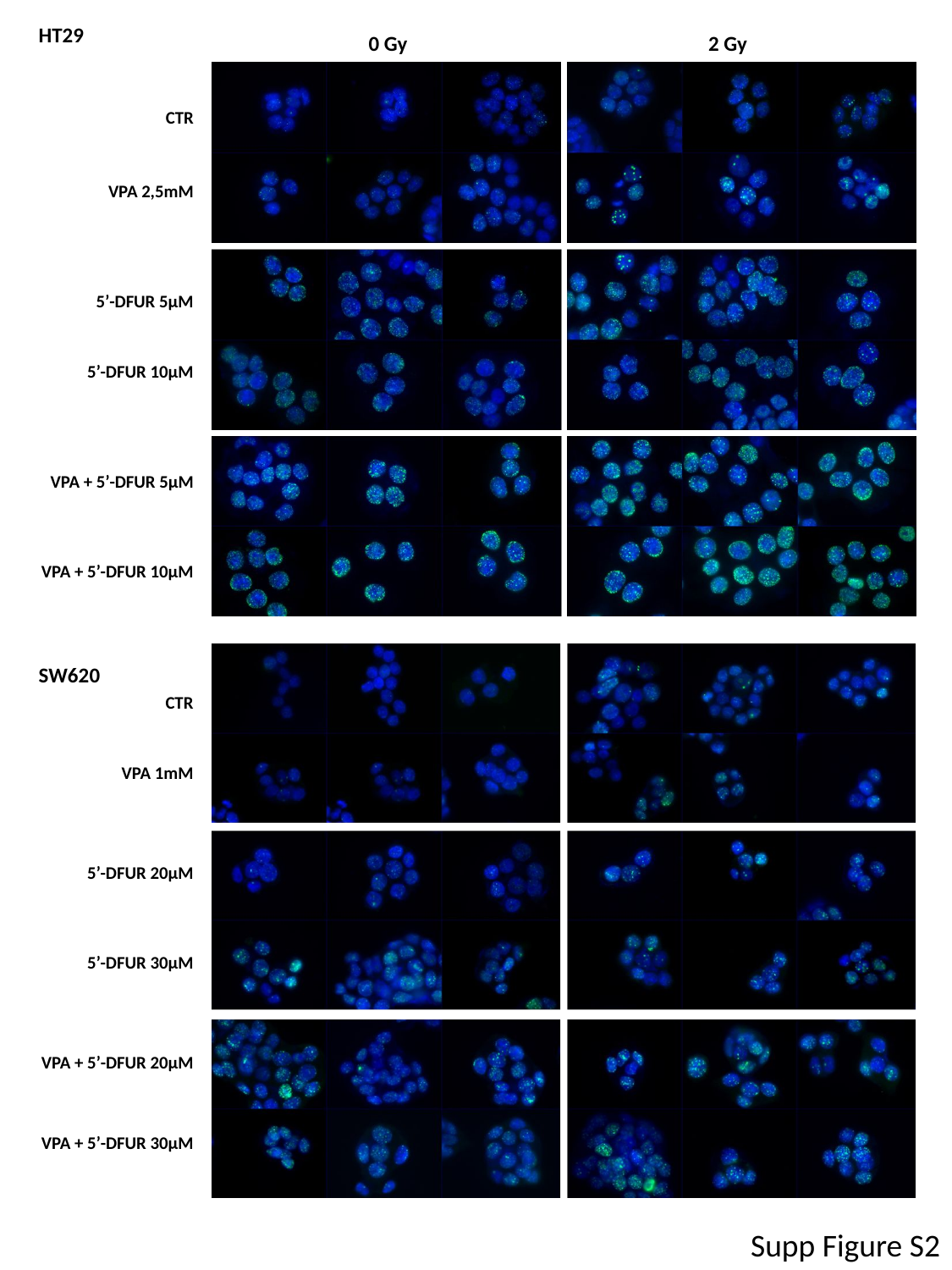

HT29
0 Gy
2 Gy
CTR
VPA 2,5mM
5’-DFUR 5µM
5’-DFUR 10µM
VPA + 5’-DFUR 5µM
VPA + 5’-DFUR 10µM
SW620
CTR
VPA 1mM
5’-DFUR 20µM
5’-DFUR 30µM
VPA + 5’-DFUR 20µM
VPA + 5’-DFUR 30µM
Supp Figure S2
