# Supplementary figures and images for: Synergistic antitumor interaction between valproic acid, capecitabine and radiotherapy in colorectal cancer: critical role of p53
Source: J Exp Clin Cancer Res. 2017 Dec 6;36:177. doi: 10.1186/s13046-017-0647-5 (PMC5719792; doi:10.1186/s13046-017-0647-5)

## Slide 1
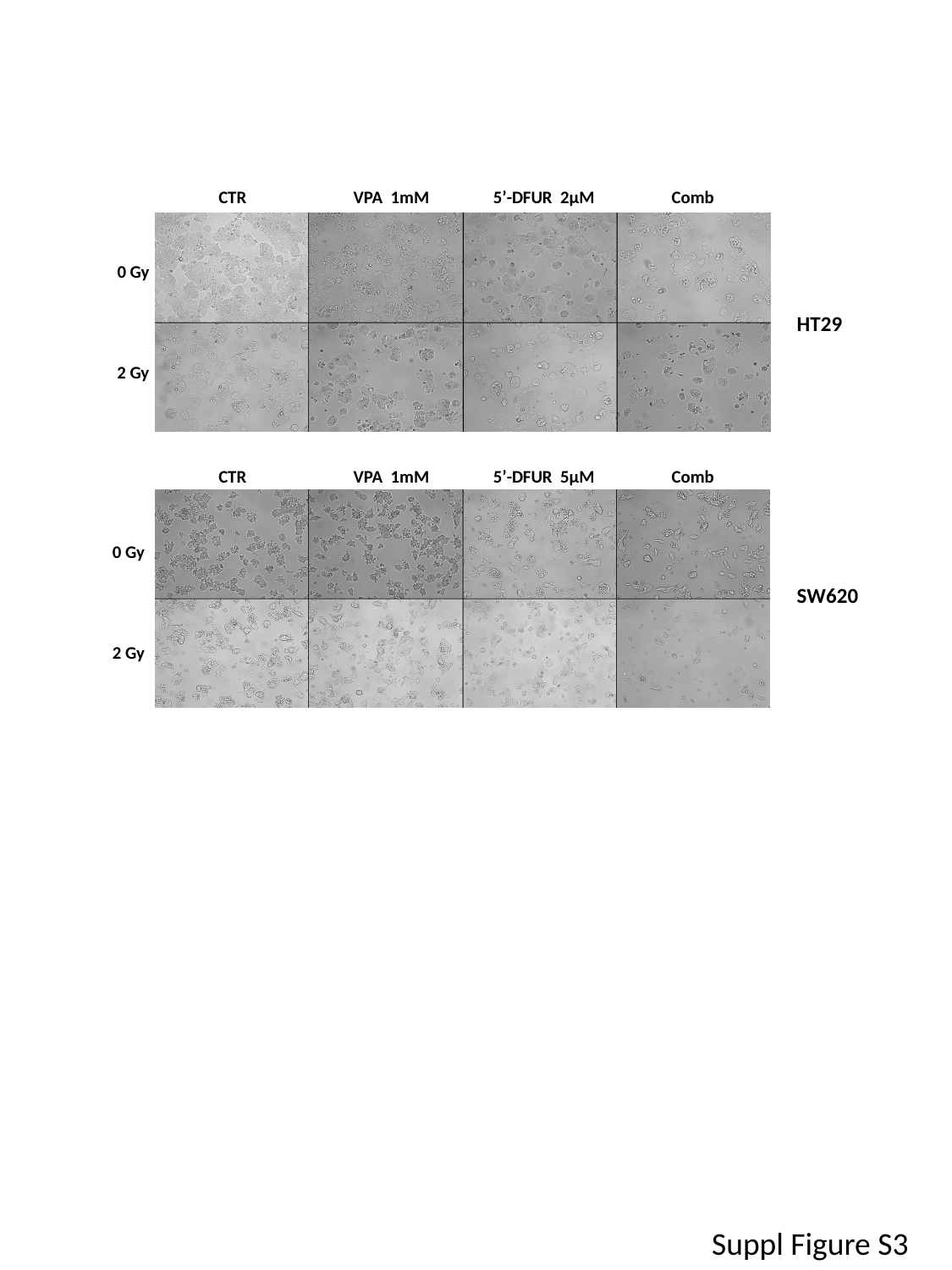

CTR
VPA 1mM
5’-DFUR 2µM
Comb
0 Gy
HT29
2 Gy
CTR
VPA 1mM
5’-DFUR 5µM
Comb
0 Gy
SW620
2 Gy
Suppl Figure S3

Supplement: Supplementary file 3 — HT29 and SW620 cells were treated or untreated with VPA 1 mM and 5′-DFUR at the indicated concentration, corresponding to IC30 at 96 h for 24 h followed or not by 2 Gy RT. Morphology and survival were examined after 48 h and phase contrast images of representative area are showed. A photograph of one well in a representative experiment is shown for each treatment. (PPT 6438 kb) [file 13046_2017_647_MOESM3_ESM.ppt]
